# Supplementary material for: Examining the Effects of the RUNX1 p.Leu43Ser Variant on FPD/AML Phenotypes Using a CRISPR/Cas9-Generated Knock-In Murine Model
Source: Biomolecules. 2025 May 12;15(5):708. doi: 10.3390/biom15050708 (PMC12109519; doi:10.3390/biom15050708)
Supplement: Supplementary file 1 [file biomolecules-15-00708-s001.zip › biomolecules-3595940-supplementary.pdf]

Supplementary Materials

Table S1. Top 50 deregulated genes.

Gene	RUNX1$^{WT/WT}$	RUNX1$^{WT/WT}$	RUNX1$^{WT/WT}$	RUNX1$^{WT/L43S}$	RUNX1$^{L43S/L43S}$	RUNX1$^{L43S/L43S}$	Het#1	Hom#1	Hom#2
Ppp1r42	0.558906	0.289661	0.077168	-0.252448	2.205.372	-0.056555	-1.013.067	-0.856777	-0.95226
Dst	-0.583506	-0.78697	-0.636148	1.084.784	-0.515731	-0.73115	-0.123486	0.14215	2.150.056
Ctla4	-0.371183	-0.409479	-0.479272	-0.351053	-0.44979	-0.364074	-0.13732	2.642.249	-0.080078
Nrp2	-0.573635	-0.506295	-0.681789	0.712352	-1.013.037	-0.747846	0.660269	2.102.035	0.047947
Cxcr1	0.201482	2.050.578	-0.149377	-0.838894	1.216.819	-0.687824	-0.599104	-0.555802	-0.637878
Gpr55	-0.485263	-0.886883	-0.763874	-0.109389	-0.932528	-0.549356	0.88392	0.981576	1.861.796
Rbm44	-0.820427	-0.735174	-0.897398	0.426836	2.386.037	0.122353	-0.099055	-0.252995	-0.130178
Rgs2	0.185304	0.632017	0.54915	-0.440512	2.050.178	-0.154953	-0.599786	-1.201.019	-1.020.378
Cr2	0.632148	2.181.269	-0.161538	0.398463	-0.147095	-0.113613	-0.838243	-0.885053	-1.066.337
Cubn	-0.404638	-0.432632	-0.364417	-0.264588	-0.458001	-0.338003	-0.207862	2.654.274	-0.184132
Etl4	-0.501469	-0.500305	-0.469805	0.080866	-0.591281	-0.486943	-0.507882	2.487.301	0.489518
Il1f9	-0.162715	0.745365	0.45509	-0.598517	2.154.401	-0.065421	-0.623128	-0.908045	-0.99703
Rapgef1	-0.590348	-0.50162	-0.512481	-0.490053	-1.005.928	-0.409493	0.272892	2.067.876	1.169.154
Fmn1	-0.679122	-0.660874	-0.677919	1.380.197	-0.896373	-0.88091	1.364.395	1.135.456	-0.084851
Slpi	0.05754	0.82818	0.875017	-0.733171	1.655.685	0.357956	-1.191.963	-0.9861	-0.863144
Sirpb1b	0.440795	1.185.963	0.407285	-0.413213	1.739.417	-0.480386	-1.075.222	-0.798009	-1.006.629
Sirpb1c	0.218195	1.154.188	0.736047	-0.021334	1.540.075	-0.448419	-0.890645	-0.927088	-1.361.018
Gm5150	0.327173	0.844059	0.365137	-0.46834	2.062.869	-0.514319	-0.785272	-0.83077	-1.000.536
S100a6	-0.102228	0.264322	0.287327	-0.583242	2.426.874	-0.118292	-0.699084	-0.826481	-0.649196
S100a11	0.439489	0.937197	0.745634	-1.023.923	1.436.282	0.430818	-1.200.231	-1.078.817	-0.686449
Rhoc	-0.279139	0.846896	0.902028	0.253875	1.637.207	-0.09767	-1.327.467	-0.94481	-0.99092
Tnfrsf8	-0.689166	-0.328437	-0.837714	0.153972	-0.952293	-0.434948	2.297.369	0.44435	0.346865
Steap4	-0.091836	1.062.987	-0.167527	-0.404712	2.221.023	-0.607337	-0.622976	-0.779505	-0.610116
Parm1	-0.469106	-0.723601	-0.491563	-0.672556	-0.99111	-0.392903	0.913843	1.958.769	0.868228
Gpat3	0.250134	0.9378	0.308419	-0.72927	1.850.943	0.293821	-1.175.278	-0.989891	-0.746677
Acacb	-0.639302	-0.686634	-0.638757	-0.115029	-0.89515	0.036334	1.846.076	1.518.661	-0.426199
Myl2	0.49745	0.545195	0.719258	-0.882953	1.671.849	0.436333	-0.923	-0.723319	-1.340.814
Asprv1	-0.539831	1.064.246	0.771125	-0.678382	1.876.715	-0.046691	-0.798497	-0.834521	-0.814164
Gm1965	-0.568492	-0.704696	-0.589107	-0.11333	-0.810972	-0.396216	138.458	2.010.823	-0.21259
Clec4d	1.614.632	0.759651	-0.318417	-0.825021	1.430.165	-0.346878	-0.991108	-0.588524	-0.734501
Clec7a	0.29636	0.910805	0.187983	-0.209559	2.014.219	-0.555306	-1.214.199	-0.980429	-0.449875
Klra17	0.068612	0.908421	-0.181225	0.0222	2.192.296	-0.594916	-0.918219	-0.668712	-0.828456
Klra1	1.931.474	0.907966	-0.163794	-0.401343	-0.061993	0.645428	-0.690527	-1.007.938	-1.159.272
Ncr1	1.595.008	1.098.288	0.907605	-0.425993	-0.751007	0.184277	-1.130.822	-1.110.485	-0.36687
Ceacam10	-0.267766	0.700753	0.983818	-0.670362	1.874.816	0.109807	-0.877483	-0.876254	-0.97733
Mrgpra2b	0.028811	0.856532	1.090.924	-0.886261	1.324.563	0.591708	-1.156.845	-0.66311	-1.186.322
Dnajb13	-0.643361	-0.776472	-0.553134	-0.065198	-0.801178	-0.567556	0.181314	1.126.023	2.099.562
1600010M07Rik	0.82756	1.047.546	0.656525	-0.35917	1.232.192	0.012445	-1.361.713	-0.733798	-1.321.586
Bnip3	0.932248	0.77917	0.801365	-0.083074	1.204.163	-0.000802	-1.198.942	-1.105.801	-1.328.328
Fcer2a	1.458.201	-0.241552	0.723287	0.463469	-0.010437	1.016.579	-0.813037	-1.221.256	-1.375.252
Tmem38a	-0.105109	0.595138	1.487.806	-0.271705	1.273.925	0.268631	-1.267.617	-0.786633	-1.194.437
Hmgcll1	1.914.651	0.662582	-0.258402	-0.580037	0.001482	0.987723	-0.909908	-1.042.504	-0.775588
Slco2a1	-0.459979	-0.448145	-0.410022	-0.364777	-0.471128	-0.392415	0.02382	-0.103964	262.661
Fyco1	-0.80163	-0.696537	-0.765406	1.006.844	-0.864533	-0.772183	0.400404	1.821.416	0.671626
Prdm1	-0.296337	-0.559196	-0.665289	-0.283206	-0.758095	-0.600061	-0.259345	13.782	2.043.329
Vpreb3	1.556.189	0.492794	0.015502	-0.650973	-0.400275	1.554.576	-0.886471	-1.100.684	-0.580657
Cpm	1.212.029	1.203.126	0.023148	-0.532268	-0.449596	133.834	-1.018.871	-119.724	-0.578668
Gm12057	1.932.122	0.561999	0.23489	-0.34299	-0.443741	0.898229	-1.064.918	-0.708748	-1.066.842
Tmem132e	1.975.742	-0.040734	0.080088	-0.412917	-0.831655	1.324.645	-0.66919	-0.772202	-0.653777
Heatr9	-0.382836	-0.633734	-0.599	-0.357446	-0.526891	-0.559485	-0.304556	1.123.502	2.240.446

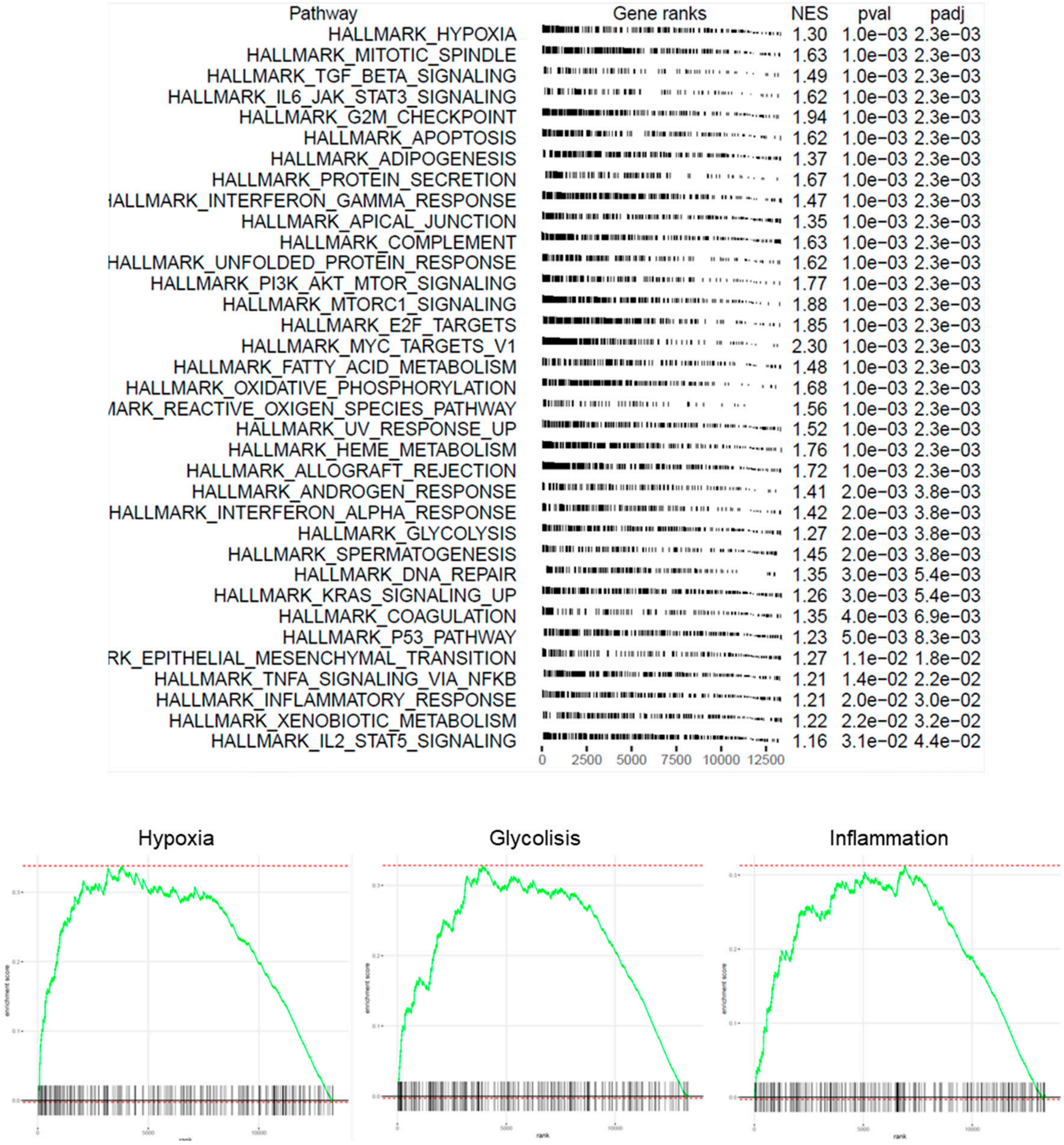

Figure S1. Enrichment score using FGSEA analysis revealed, among others, deregulated genes involved in hypoxia, glycolysis, or inflammatory response.

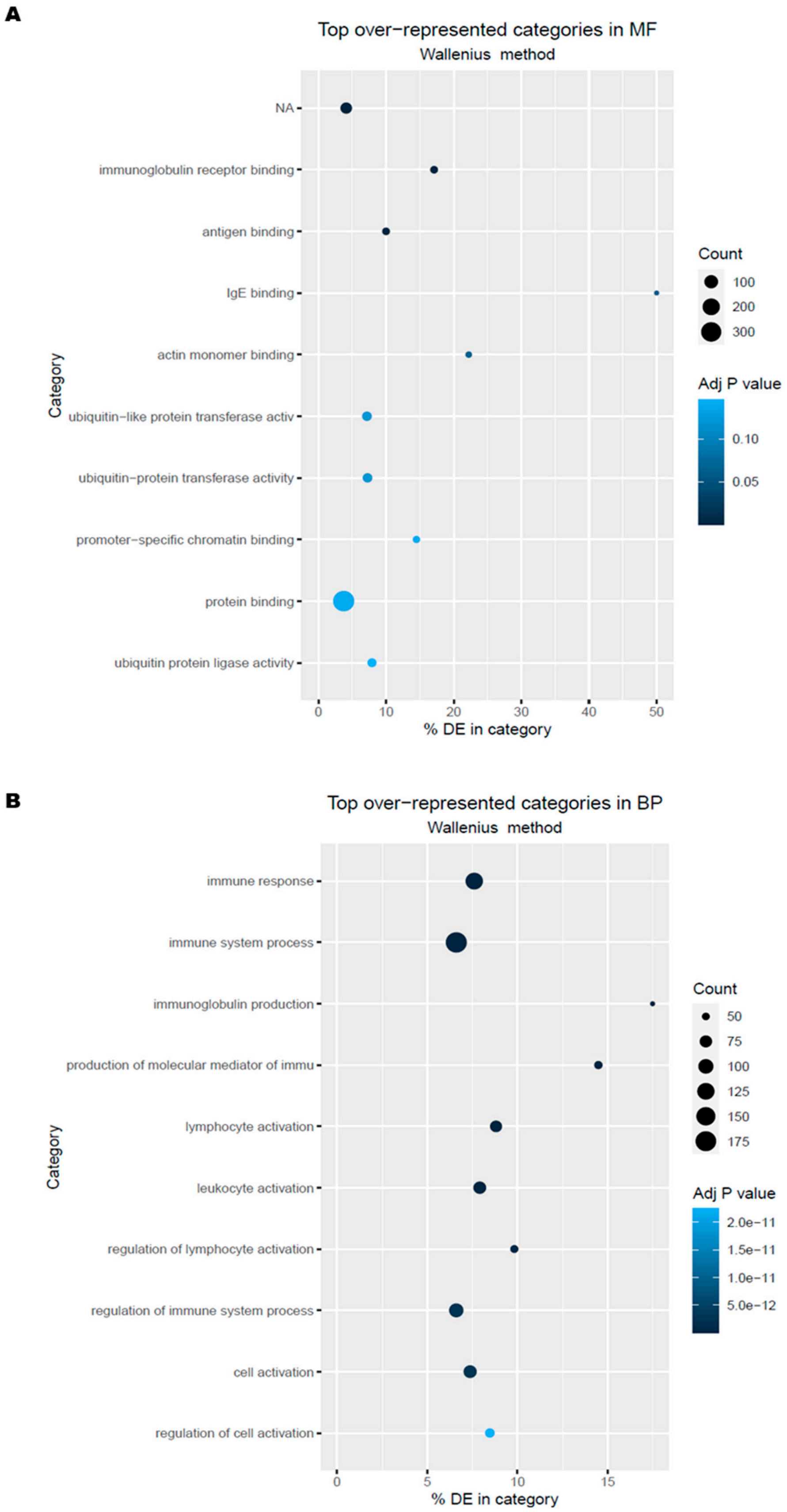

Figure S2. Top over-represented categories using the Wallenius method. (A) Regarding molecular function. (B) Biological processes.
